# The variety of clinical presentations in IgG4-related disease in Rheumatology

**DOI:** 10.1007/s00296-017-3807-1

**Published:** 2017-08-30

**Authors:** Agata Sebastian, Maciej Sebastian, Maria Misterska-Skóra, Piotr Donizy, Agnieszka Hałoń, Arkadiusz Chlebicki, Artur Lipiński, Piotr Wiland

**Affiliations:** 1 Department of Rheumatology and Internal Medicine, Wroclaw Medical Hospital, Borowska 213, 50-556 Wroclaw, Poland; 2 Department of Minimally Invasive Surgery and Proctology, Wroclaw Medical Hospital, Borowska 213, 50-556 Wroclaw, Poland; 3 Division of Pathomorphology and Clinical Cytology, Department of Pathomorphology, Wroclaw Medical University, Borowska 213, 50-556 Wroclaw, Poland; 4 Department of Rheumatology and Internal Medicine, Wroclaw Medical University, Borowska 213, 50-556 Wroclaw, Poland

**Keywords:** IgG4, Treatment, Differential diagnosis, Pathology

## Abstract

IgG4-related disease (IgG4-RD) belongs to the group of rare diseases in which the identification of the characteristic histology and immunohistochemistry provides with the gold standard in the diagnosis. The variable organ dysfunction reflects the clinical presentation. The examples of different IgG4-RD presentations in the Rheumatology Unit were discussed in this article. The spectrum of IgG4-RD is wide-ranging and manifested in one or more organs synchronously or metachronously. In the presented article, we described five different cases of IgG4-RD. Four cases were reaffirmed in the histopathological assessment. The clinical and laboratory findings were analyzed and the assigned therapy was discussed. According to our experience, the diagnosis of IgG4-RD requires the careful clinicopathological correlation. The diagnosis relies on the coexistence of various clinical, laboratory, radiological, and histopathological findings, although none of them is pathognomonic itself. The time needed for the diagnosis and variety of clinical forms of IgG4-RD shows that there is need of the cooperation among many specialists for the better and earlier recognition of the disease.

## Introduction

The IgG4-related disease (IgG4-RD) is a chronic, inflammatory, multi-organ, systemic disease [1]. The name was first proposed by the Japanese investigators in 2010 [2], and the comprehensive diagnostic criteria for the IgG4‑RD were first determined and unified by Umehara et al. in 2011 [3]. The disease can affect many organs: most commonly the pancreas, liver, bile ducts, thyroid gland, aorta, retroperitoneum, lymph nodes, lacrimal glands, and occasionally the brain [4]. The variable organ dysfunction reflects the clinical presentation. Patients may be asymptomatic and only incidentally diagnosed at the physical examination or imaging. They may present a single or multiple organ involvement. The histopathological assessment is the diagnostic gold standard, hallmarked by the lymphoplasmacytic infiltrates of IgG4-plasma cells, the storiform fibrosis, tissue eosinophilia, and the obliterative phlebitis. Large epidemiologic studies are lacking. The exact frequency of IgG4-RD in Europe is still unknown. The prevalence of IgG4-RD in the Rheumatology Units is low, the diagnostic process is time-consuming, it may mimic other diseases and there is need for the specific histological assessment.

This article gives examples of different IgG4-RD presentations in the Rheumatology Unit in Eastern Europe.

## Materials and methods

The patients were diagnosed with IgG4-RD in the Rheumatology and the Internal Medicine Department between 2014 and 2016. The data were collected retrospectively. The inclusion criteria were: age >18 years and the histopathological picture of IgG4-RD. The data included the time from the first symptoms to the establishment of IgG4-RD diagnosis, the age and gender of patients, the localization of lesions, histopathological biopsies, IgG4, IgG, and IgM concentration in blood samples, the erythrocyte sedimentation rate (ESR) and C-reactive protein (CRP) values, C3 and C4 values treated with and the responsiveness to corticosteroids (CS), and the history of the atopic disease. Patients with the different IgG4-RD presentations were chosen to illustrate the complexity of IgG4-RD cases treated in the Rheumatology Unit. We performed the literature review following the methods specified by Gasparyan et al. [5]. The searches were conducted in the PubMed (part of the National Library of Medical Databases) and Cochrane Library. The search strategy was based on the publication since 2010. The key word included: IgG4 related disease or IgG4 or autoimmune pancreatitis or Mikulicz disease. Authors analyzed the review articles and case reports.

## Results

**Fig. 1:**
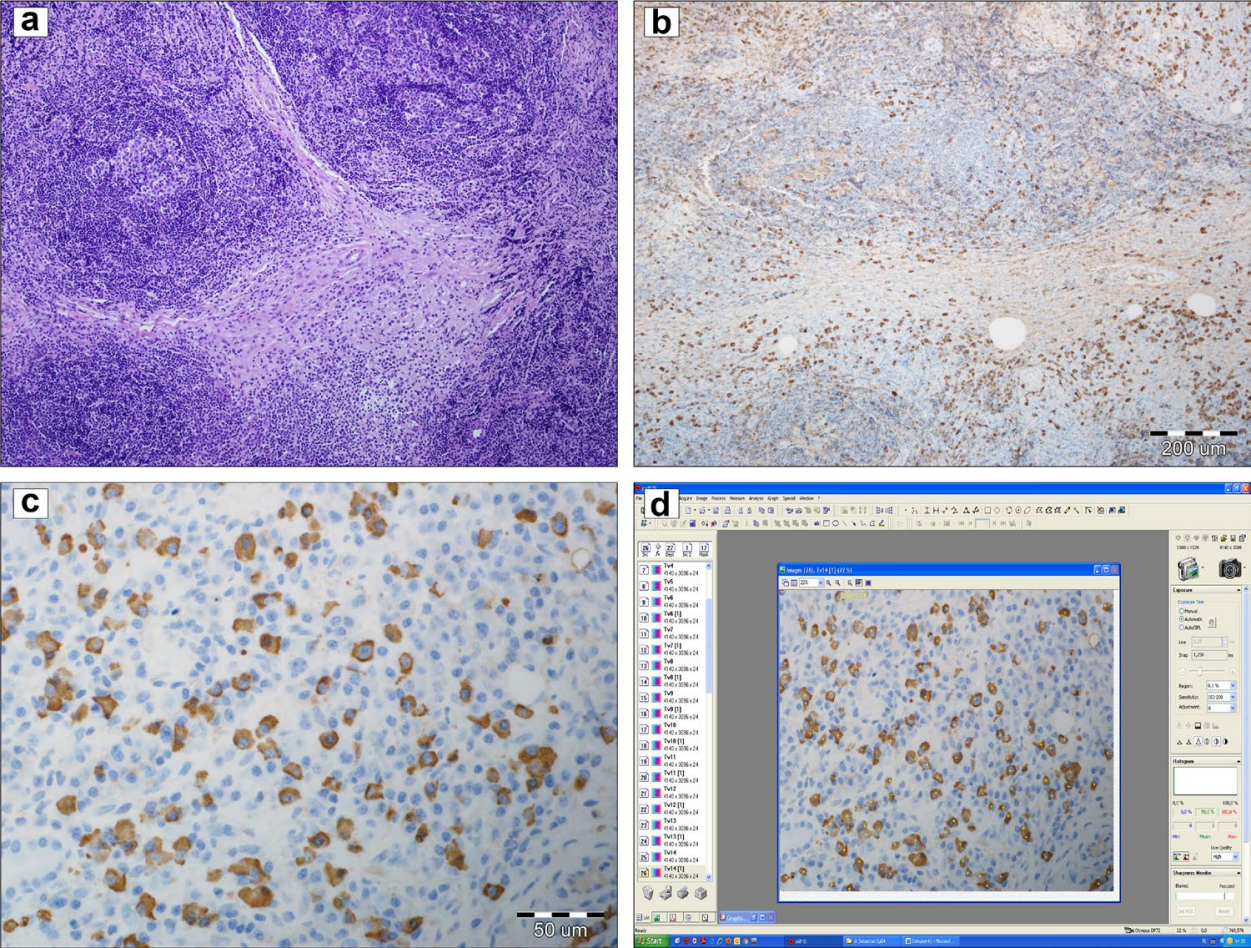
The lacrimal gland with the dense lymphoplasmacytic infiltration and advanced fibrosis, which are highly histologically suggestive for IgG4-related disease (**a** 200×, H&E staining). The inflammatory infiltration in the presented above lacrimal gland is predominantly consisted of the IgG4-positive plasma cells (**b** 200×, hematoxylin). Immunostaining for the IgG4 in the salivary gland in one high-power field (hpf), which is an accepted method for counting of the IgG4-positive plasma cells based on Deshpande et al. [6] (**c** 400×, hematoxylin). The increased number of IgG4-positive plasma cells in the salivary gland (**d**): 112 IgG4-positive plasma cells based on the detailed counting with Cell^D Program (Olympus, Poland)

**Fig. 2:**
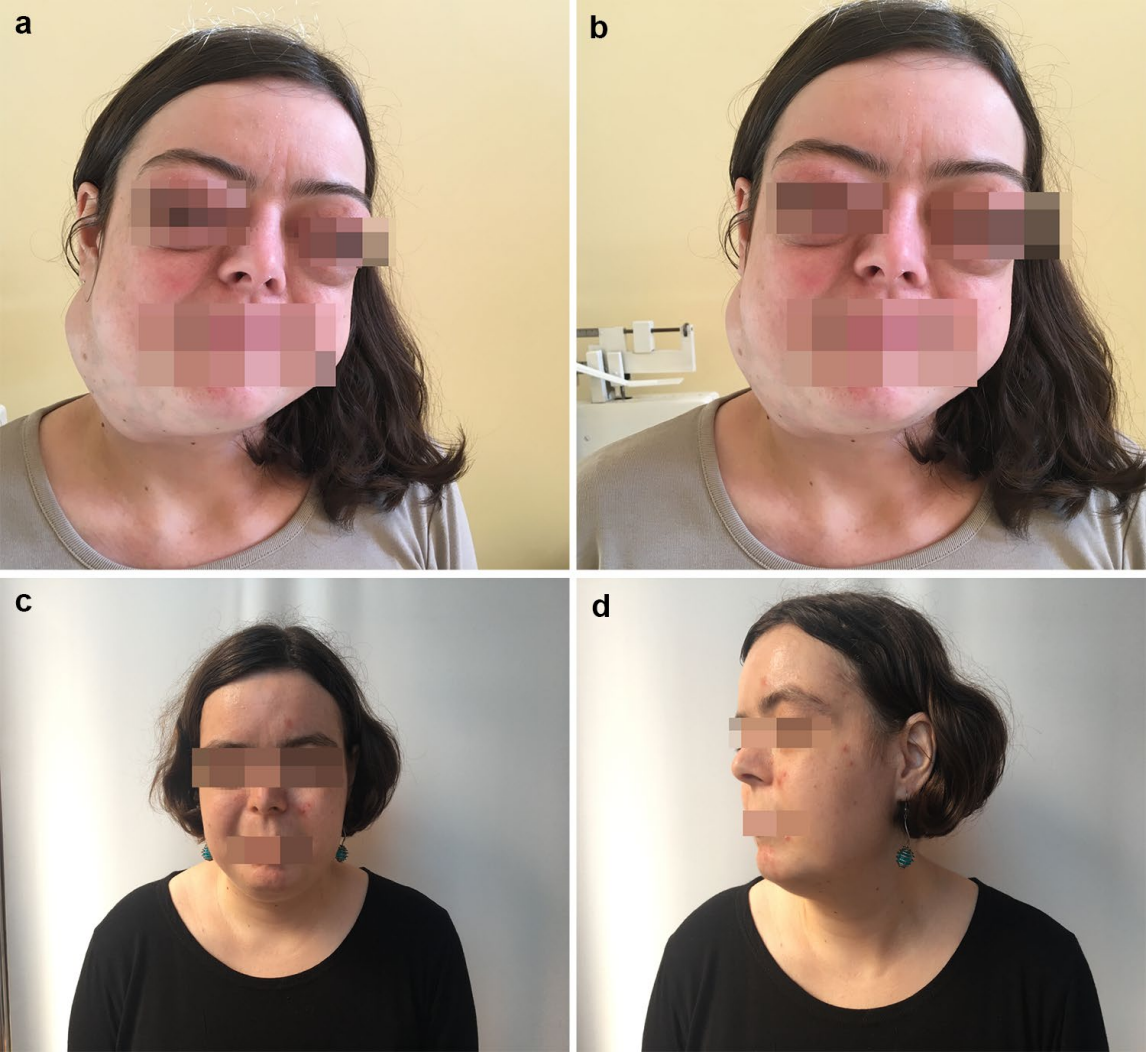
36-year-old female patient. The characteristic changes of IgG4-RD (**a** and **b**). It is the typical Mikulicz disease (MD) with the lacrimal glands enlargement and salivary glands enlargement which deformed normal features. The improvement after 3 weeks of the treatment with glucocorticoids (**c** and **d**)

**Fig. 3:**
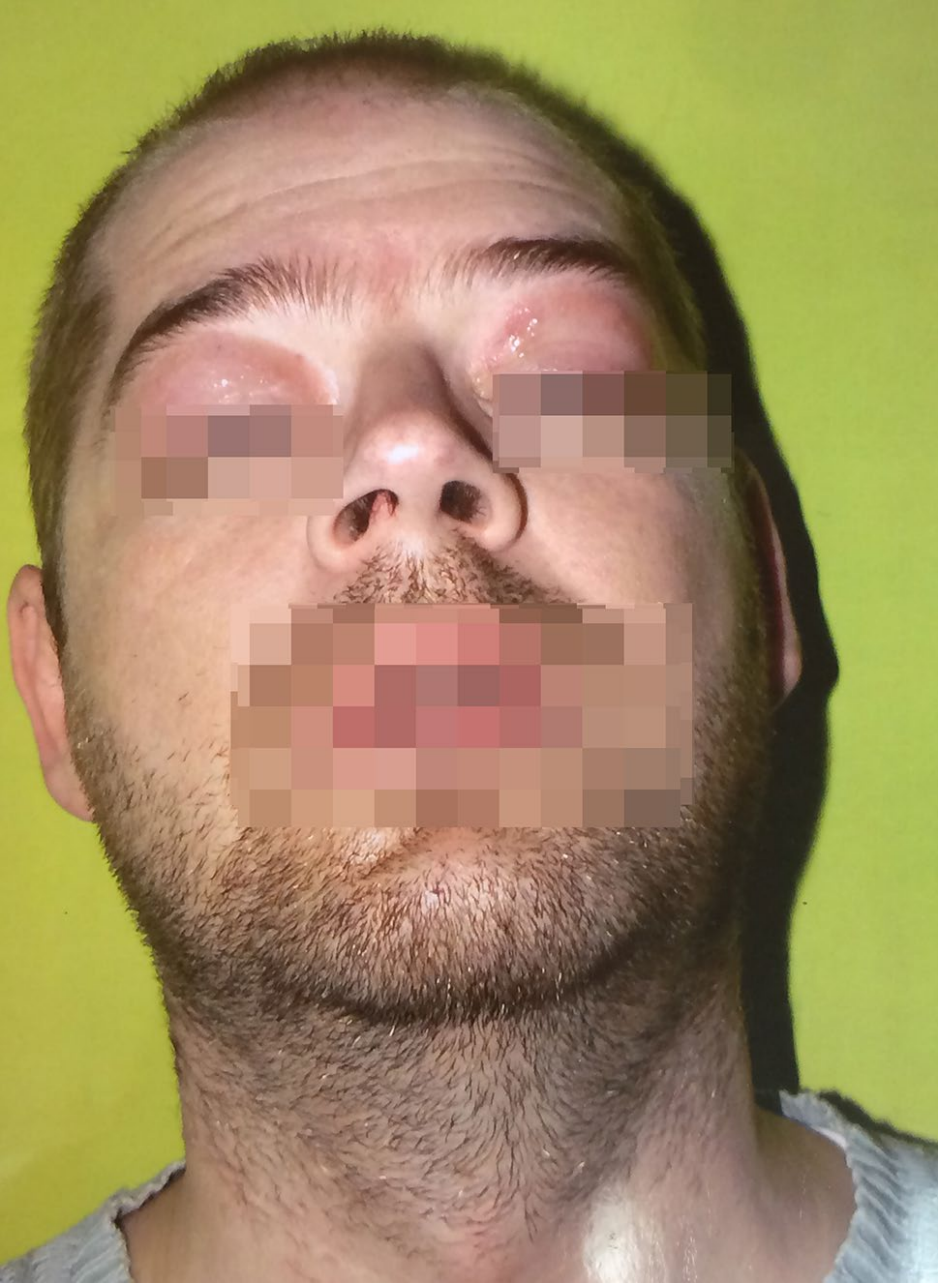
The lacrimal gland enlargement in the patient with the simultaneously diagnosed ileus pseudotumor

Five cases of IgG4-RD were diagnosed. Four cases were reaffirmed in the histopathological assessment (Fig. 1). The main localization of IgG4-RD was the salivary glands (four cases). In two cases, the lacrimal gland enlargement was the first presentation of IgG4-RD (Fig. 2). In one case, it was the unilateral submandibular gland enlargement with the granulomatosis and polyangiitis (GPA) found in the biopsy. In case number four the pancreatitis was the first clinical presentation of IgG4-RD. The suspicion of IgG4-RD was made due to the increased IgG4 serum concentration, eosinophilia, concomitant parotid, the submandibular glands enlargement, peripheral lymphadenopathy, and the lack of specific antibodies, e.g., antinuclear antibodies. In case number five, the clinical presentation included the lacrimal enlargement, peripheral lymphadenopathy, and the clinical signs of ileus (Fig. 3). The characteristics of the clinical symptoms of the disease are demonstrated in Table 1 and Figs. 2, 3. None of the patients had the positive symptoms of dryness or the allergy history. Antinuclear antibodies (ANA) were positive only in case number one, but in a very low titer. The hypocomplementemia C3 and C4 were observed in case number three, where IgG4-RD and GPA were diagnosed. Three patients had the elevated blood IgG4 concentration, but not IgG fraction except case number three. In this case, the IgG4 fraction was not assessed because IgG4-RD was not suspected at the beginning of the diagnostic process. In the cases with the lacrimal gland enlargement, the IgM fraction in the serum was decreased. ESR and CRP concentrations were elevated in two patients. The laboratory findings of the patients with IgG4-RD are presented in Table 2. The therapy with CS was introduced in all the cases. In case number three, methotrexate was added due to the concomitant vasculitis. In case number five, mycophenolate mofetil was added due to the partial response to CS therapy and then changed into methotrexate due to leukopenia.

**Table 1:** Clinical characteristics of patients with IgG4-RD in rheumatology unit

Number of patient	1	2	3	4	5
Age (years)	36	43	59	42	34
Gender	Female	Male	Male	Male	Male
First manifestation	Lacrimal glands enlargement	Lacrimal glands enlargement	Unilateral parotid gland enlargement	Pancreatitis	Ileus
Localization	Parotid and lacrimal glands	Parotid and lacrimal glands	Parotid glands, peripheral lymphadenopathy, mucosal ulcerations, weight lose	Parotid and submandibular glands, pancreas, (diabetes mellitus t2), peripheral lymphadenopathy	Ileus, lacrimal glands, lymphadenopathy
Proven in histopathology	Yes-lacrimal glands	Yes-lacrimal glands	Yes-salivary glands + GPA	No	Yes
History of allergic diseases	No	No	No	No	No
Glucocorticoid response	Yes	Yes	Yes + MTX 15 mg/w p.o.	Yes + AZA 100 mg/day	Partial-(MMF leukopenia), MTX 15 mg/week
Time to IgG4-RD diagnosis (months)	132	72	4	48	17

**Table 2:** Laboratory findings among patients with IgG4-RD

Number of patient	1	2	3	4	5
IgG4 in serum (*n* 0.11–1.57 g/l)	3.77	3.83	Not done	3.35	0.54
IgG in serum (*n* 7–16 g/l)	10	11.76	48.14	10.7	6.1
IgM in serum (*n* 0.4–2.3 g/l)	0.25	0.28	0.76	1.8	0.68
ESR mm/hr	39	2	86	10	8
CRP (*n* 0–5 mg/l)	19.1	2.61	14.6	2	2.35
C3 (*n* 0.9–1.8 g/l)	1.93	1.4	0.57	1.0	0.65
C4 (*n* 0.1–0.4 g/l)	0.76	0.32	0.08	0.4	0.18
Eosinophilia in blood	No	No	No	Yes	No
ANA dilution	320 (specific-no)	No	No	No	Negative

*GPA* granulomatosis with polyangiitis, *MTX* methotrexate, *AZA* azathioprine, *MMF* mycophenolate mofetil, *n* normal value

## Discussion

IgG4-RD belongs to the group of rare diseases. Three major histopathological features of IgG4-RD are: the lymphoplasmacytic infiltrates, storiform-type fibrosis of the irregularly whorled pattern resembling that of a straw mat, and the obliterative phlebitis. Sometimes the infiltration of eosinophils is observed in the affected tissues. Some lesions may be described as pseudotumors as in case number five with the first clinical presentation of ileus. Necrosis and granuloma are not found in IgG4-RD. When they are observed, the rheumatologists should think about the different diagnosis or alternative disease [6]. The diagnostic scheme of IgG4-RD includes the number of IgG4+ plasma cells in the affected tissue, and it depends on the localization and the type of biopsies (a needle biopsy or a surgical specimen) [6]. Important is that not all the histopathological features are observed in all the tissues and the histologically highly suggestive form of IgG4-RD or the probable features of IgG4-RD are distinguished [6]. The second step of the histopathological assessment is to determine the ratio of IgG4+ plasma cells to the general number of IgG cells, which should be more than 40%, except for the aorta where the ratio should be more than 50% [6]. The differential diagnosis includes lymphoma, tumor, vasculitis, and abscess and, therefore, the alternative diseases should be excluded at the beginning of the assessment. IgG4-RD may mimic the reactive lymphoid infiltrates and lymphoma, particularly MALT lymphoma. Both clinically and pathologically, IgG4-RD is manifested as the mass-forming lesion composed of the dense lymphoplasmacytic infiltrate [7–9]. IgG4-RD with the concomitant vasculitis, e.g., with GPA as in case number three, is rarely observed. In this patient, the clinical symptoms were more severe than in the other patients with the weight loss and mucosal ulcers in the nose and mouth.

In the non-biopsy-proven cases, e.g., the pancreas or brain localization, the measurement of the IgG4 concentration in blood samples is useful. Based on IgG4-RD definition, it should be elevated above 135 mg/dl [10] and it is observed in most patients. Nevertheless, 20–40% of the patients with IgG4-RD may have the IgG4 value in the normal ranges. On the other hand, the increased amount of IgG4 can be observed in different diseases: the primary Sjögren syndrome (7%), lupus erythematosus (10%), and rheumatoid arthritis, cancers and also in the healthy population (2%), but the criteria of IgG4-RD are fulfilled only by individuals [10, 11]. The IgG4 production depended on, among others, Il-10 and Il-6. Among 136 patients with the rheumatoid arthritis, the increased level of IgG4 in the serum was observed in 46% of patients and was correlated with the active disease [11].The elevated serum IgG4 levels were the most important laboratory features of IgG4-RD both in our and other publications [12, 13].The abnormal C3 and C4 complement levels were not observed in the presented cases, except the case with vasculitis, and they are not typical for IgG4-RD, but more frequently appear in other autoimmune disorders.

In one patient we observed eosinophilia, which occurs in one-third of the patients with IgG4-RD like the allergy history according to the recent publications [13]. Yu Chen et al. reported a higher frequency of allergic diseases (59%), but this was not correlated with the increased IgE serum concentration which was found in 83% of patients. In addition, the correlation between eosinophilia (33% of patients) and the allergy history was not observed [13]. The history of the atopic disease included, for example, the bronchial asthma, allergic rhinitis, nasal polyps, and atopic dermatitis [14]. In our group of patients, we did not observe this correlation between IgG4-RD symptoms and the history of allergic diseases. The IgE serum concentration was not measured because it was technically impossible.

The spectrum of IgG4-RD is wide-ranging, occurring in one or more organs synchronously or metachronously. Moreover, the patients with IgG4-RD usually do not have the constitutional symptoms, such as fever, malaise, night sweats, or the weight loss [15]. Malignancies were reported in 7.4% of patients with IgG4-RD [16].The extranodal marginal zone B cell lymphoma may occur in the ocular area, salivary glands or the meningeal dura up to 5 years after the diagnosis of IgG4-RD [17–19]. In our cohort, all of the patients had more than one organ involved. The most frequent localization was the head and neck area and the parotid glands. Two patients had the typical Mikulicz disease (MD) with the lacrimal gland enlargement. The orbital involvement in IgG4-RD is common and the orbit was the first extra pancreatic localization to be reported in the literature [20]. In the publication, MD was considered to be a subtype of IgG4-RD. In addition, it had a number of differences compared to the typical Sjögren syndrome, including not only the variety of the gender distribution (MD occurs in both men and women, while Sjögren syndrome occurs mainly in women) but the normal or mild salivary secretion dysfunction in MD [21]. The symptoms of dryness were not presented in our cohort and none of the patients met the criteria for the primary Sjögren syndrome (including the lack of the specific antibodies and normal salivary flow).

Because of the similarity to Sjögren syndrome (the lacrimal and salivary glands involvement, case numbers one to four), the diagnosis of the IgG4-RD was postponed for an average of 5 years (64 months). Generally, the clinical signs depended on the localization and organ damage caused by the main infiltration of IgG4 cells. They often formed pseudotumors as in patient number five. Nonetheless, the time for IgG4-RD diagnosis was 17 months late until the patient was admitted to the Rheumatology Unit. The shortest time for the diagnosis of IgG4-RD was in the patient with the unilateral parotid gland enlargement and the concomitant weight loss. The fast recognition of GPA caused the patient to be admitted to the Rheumatology Department. The first suspicion of lymphoma focused the diagnosis on tumor. In the presented paper, patients had been diagnosed by specialists in the general medicine, oncologists, surgeons, endocrinologists, specialists in the travel medicine, and hematologists before they were admitted to the Rheumatology Unit. In all the cases, the first diagnoses included the oncological diseases, e.g., the lacrimal lymphoma, salivary lymphoma, bowel tumor, lung cancer or hepatoma.

In the presented case series, the Type 1 (IgG4-related) autoimmune pancreatitis was observed in one patient. It is one of the most common disease manifestations that often devolves into the endocrine and exocrine insufficiency and may complicate the CS treatment. In the described patient, we started the therapy with CS in the recommended doses, despite the diabetes mellitus, with the good clinical response and improvement in the blood glucose level. In the case number four, the pancreatic biopsy was not performed because of the technical problem. In the diagnostic parameters, the IgG4 serum concentration, abdomen ultrasound, and the computed tomography were used, and the good clinical improvement after starting the CS treatment was observed. The diagnosis was ultimately made based on the classification criteria proposed by the Japanese Pancreas Society Revision in 2006 and Mayo Clinic [27–29].

The anti-nuclear antibodies were detected only in one case, but in a very low titer, without any specific antibodies, which is very typical for IgG4-RD compared to other autoimmune diseases. When the antibodies against the specific antigenes are present, the concomitant different disease is the most probable reason. The polyclonal hypergammaglobulinemia will not help in the diagnostic parameters; it is typical for Sjögren syndrome, but was often observed in IgG4-RD too [12].

Lymphadenopathy was very common in IgG4-RD patients, as in the paper of Chen [13], and occurred in most of the patients in the presented article.

In our series, the inflammatory markers ESR and CRP were elevated in two cases. The results were comparable with the other authors [12, 13, 22], who indicated that inflammatory factors did not play crucial role in IgG4-RD pathogenesis and its progression.

In the present study, all the patients were treated with the oral CS in the dose of prednisone of 0.6 mg/kg of the body weight/day. The response to this therapy was good, with the clinical and laboratory improvement. Nevertheless, in the previous publications, among the patients remaining on the long-term steroid therapy, the relapse was common after the cessation of CS [23]. Unfortunately, a large percentage of patients with this condition have the relative contraindications to the prolonged CS treatment, even in the moderate to low doses (for example, obesity, the glucose intolerance, hypertension, and osteoporosis) [24, 25]. Other therapies, such as radiation or the immunomodulating agents, are not well-described in the literature [26]. The paucity of data regarding the use of the disease-modifying anti-rheumatic drugs (DMARDs) provides with a little support for the use of these medications in the clinical practice, although they are often used in the hope of reducing the CS dependency. For this reason, DMARDs were added in patient numbers three, four, and five due to the partial responsiveness to the CS therapy. In one case (patient number five), who was treated with the mycophenolate mofetil, leukopenia was observed after 2 months of the treatment and the medication was changed into methotrexate. More recently, the B cell depletion has shown the evidence of the CS-sparing efficacy and has even been used as a monotherapy, with considerable success [24], but this therapy option is not commonly available in Eastern Europe.

There are some limitations of this study. First of all, there were a small number of patients enrolled only in one Rheumatology Unit. The time for the diagnosis of IgG4-RD was relatively long. In the presented cases, it was the mean of 5 years, suggesting that multicenter prospective study is needed to evaluate the clinical and laboratory features of IgG4-RD more broadly. Moreover, the benefits of the diagnostic methods in the diagnosis of IgG4-RD in the Rheumatology Units should be evaluated in a larger amount of patients. In our experience, the diagnosis of IgG4-RD requires the careful clinicopathological correlation. The diagnosis relies on the coexistence of various clinical, laboratory, radiological, and histopathological findings, although none of them is pathognomonic itself. The time needed for the diagnosis and the variety of clinical forms of IgG4-RD confirmed that there is a need for the cooperation among many specialists for the better and earlier recognition of the disease.
